# Highlights Regarding Host Predisposing Factors to Recurrent Vulvovaginal Candidiasis: Chronic Stress and Reduced Antioxidant Capacity

**DOI:** 10.1371/journal.pone.0158870

**Published:** 2016-07-14

**Authors:** Luciene Akimoto-Gunther, Patrícia de Souza Bonfim-Mendonça, Gisele Takahachi, Mary Mayumi T. Irie, Sônia Miyamoto, Márcia Edilaine Lopes Consolaro, Terezinha I. Estivalet Svidzinsk

**Affiliations:** 1 Postgraduate Program in Health Sciences, State University of Maringá, Maringá, Brazil; 2 Department of Clinical Analyses and Biomedicine, State University of Maringá, Maringá, Brazil; Institute of Microbiology, SWITZERLAND

## Abstract

We studied host factors that could predispose women to develop recurrent vulvovaginal candidiasis (RVVC), including glycemia, insulin resistance, chronic stress, antioxidant capacity, overall immune status, local inflammation and vaginal microbiota. The presence of yeasts in vaginal culture was screened in 277 women, with or without signs and symptoms of VVC and RVVC. The presence of an inflammatory process and microbiota were analyzed through vaginal bacterioscopy and cervical-vaginal cytology, respectively. Fasting-blood samples were collected by standard venipuncture for biochemical analyses. Flow cytometry was employed to obtain the T helper/T cytotoxic lymphocyte ratio, and insulin resistance was assessed by the HOMA index (HI). Yeasts were isolated from 71 (26%) women: 23 (32.4%) with a positive culture but without symptoms (COL), 22 (31%) in an acute episode (VVC), and 26 (36.6%) with RVVC. *C*. *albicans* was the main yeast isolated in all clinical profiles. The control group (negative culture) comprised 206 women. Diabetes mellitus and insulin resistance were more associated with the positive-culture groups (COL, VVC and RVVC) than with negative ones. The RVVC group showed lower mean levels of cortisol than the control group and lower antioxidant capacity than all other groups. The T Helper/T cytotoxic lymphocyte ratio was similar in all groups. The RVVC group showed a similar level of vaginal inflammation to the control group, and lower than in the COL and VVC groups. Only the CVV group showed a reduction in vaginal lactobacillus microbiota. Our data suggest that both chronic stress (decreased early-morning cortisol levels) and reduced antioxidant capacity can be host predisposing factors to RVVC.

## Introduction

Recurrent vulvovaginal candidiasis (RVVC) is a common cause of significant morbidity in women in all strata of society affecting millions of women worldwide [1,2]. Previously RVVC occurrence was limited by onset of menopause but the widespread use of hormone replacement therapy has extended the at-risk period [2]. VVC and RVVC are diseases caused mainly by members of the genus *Candida* [1–3]. These yeasts, in particular *Candida albicans*, are capable of colonizing the human body without producing signs of disease in conditions of physiological equilibrium [1,3]. However, under conditions that disrupt the delicate balance between the host and this commensal fungus, a parasitic relationship may occur, resulting in the development of infections [1–3].

Fungus virulence attributes including adhesion capacity, biofilm development, cell-surface hydrophobicity, morphological transition, and hydrolytic enzymes are related to intrinsic properties of yeasts that increase their ability to cause VVC [4,5]. A recent study showed that the higher prevalence of vaginal candidiasis among diabetics could be related to increased aspartyl proteinases production in this group of patients [6]. In addition to yeasts virulence factors, several host predisposing factors have been suggested [1–3] although few have proved to be involved in the pathogenesis of VVC and particularly RVVC.

The cellular immune response systemic or local and defective neutrophil function may increase susceptibility to infections by *C*. *albicans*, [7,8] suggesting that clinical conditions such as exposure to chronic stress, diabetes mellitus, or deficient antioxidant micronutrients that alter the proper functioning of the immune system could facilitate RVVC occurrence. Nevertheless, few data are available to help in evaluating the host factors involved in the host-fungus interaction and disease development. In order to further elucidate these relationships, we analyzed intrinsic factors that could predispose women to developing RVVC, including glycemia, insulin resistance, chronic stress, antioxidant capacity, overall immune status, presence of vaginal inflammation, and microbiota.

## Materials and Methods

This prospective study included 277 sexually active patients, aged between 18 and 50 years, who were referred to the Clinical Analysis and Research Laboratory of the State University of Maringá (UEM), Paraná, Brazil, from July 1, 2012 to June 30, 2013. The study was carried out with females who visited the laboratory to provide cervical-vaginal samples for clinical analyses, independently of the presence of VVC symptoms. Exclusion criteria were: hysterectomized, pregnant, or in post-partum condition; less than 14 years of age; some degree of difficulty in understanding the study; vaginal bleeding; sexual intercourse/vaginal douching within the 48 hours preceding the collection of the vaginal sample; those who reported receiving antibiotics, corticosteroids or immunosuppressive drugs; and also those with a history of serious debilitating or immunosuppressant disease, including AIDS. All the women or parents gave their written informed consent to participate in the study. This research was approved by the Committee for Ethics in Research with Humans (COPEP) of Universidade Estadual de Maringá (UEM) (report no. 435/2009).

After giving their consent, participants were asked to complete an enrollment questionnaire that included detailed questions regarding hypothesized risk factors of VVC (personal/sexual habits, history of VVC diagnoses during lifetime and the past year), presence of VVC signs and symptoms (vaginal discharge, vulvovaginal itching, vulvovaginal burning sensation, dysuria, and dyspareunia), and epidemiological data. Vaginal discharges were appraised by the health professional during sample collection [9].

Vaginal samples were collected with sterile swabs and a disposable vaginal speculum (Vagispec, Santa Catarina, Brazil), inoculated in sterile saline, and immediately seeded onto plates containing Sabouraud dextrose agar (SDA) (Difco, Detroit, Michigan, USA), supplemented with 50 mg/mL chloramphenicol (Sigma Aldrich, St. Louis, Michigan, USA) and incubated at 25°C for up to five days. A pool of the colonies grown on each plate was subcultured on CHROMágar *Candida* (Probac, Paris, France) to ensure the purity of the isolates and to identify mixed cultures. Beginning with the pure culture, the yeasts were identified by classical phenotypic methods [10]. The yeasts were stored in Sabouraud dextrose broth (SDB) (Difco, Detroit, Michigan, USA) with 10% glycerol at -20°C.

Women with a positive vaginal culture for yeasts were classified based on clinical profiles by physicians in three groups, as follows: 1) colonized (COL)- those without signs and symptoms of VVC; 2) with VVC- those with an acute episode, consisting of women who presented at least two signs or symptoms (discharge, itching, dysuria, and dyspareunia) but no previous episode within a 12-month period; 3) with RVVC- those presenting two or more symptoms in four or more episodes within a 12-month period [9]. Women with a negative vaginal culture were selected as the control group (CG).

A smear was prepared from the vaginal secretion for bacterioscopy. The slides were Gram-stained and examined under an optical microscope at 1.000X magnification. The stained smears were useful to evaluate the presence of yeasts and other infectious agents, vaginal microbiota, and leukocyte reaction. Vaginal microbiota was considered normal when microorganisms morphologically compatible with *Lactobacillus* spp. were predominant [10].

After sample collection for culture and bacterioscopy, cervical-vaginal samples were obtained from each participant, using an Ayre spatula and a cytobrush, in which triple smears (vaginal, cervical, and endocervical) were prepared and immediately fixed with propylene-glycol spray. The cytological smears were sent to the Clinical Cytology Laboratory (UEM) for analysis. All smears were stained with Papanicolaou (Pap), evaluated in optical microscopy and vaginal inflammation reported according to the Bethesda System/2001 diagnosis criteria by the reference cytologist [11]. The vaginal smears were analyzed for qualitative detection of cellular inflammatory changes in at least 20 different fields under optical microscopy at 400x magnification. The presence of leukocyte and cellular morphological changes in moderate or intense grade were the parameters employed to characterize the inflammation process. The main cellular inflammatory characteristics, as described by Nayar [11], are in the nucleus, that may become compact, dense, and pyknotic, with loss of all chromatin details or degenerated. Such nuclei may have a distinct circumferential cytoplasmic clearing or hollow, causing a perinuclear “halo”. The cytoplasm may show partially or completely disintegrated, amphophilia, and vacuolation [11].

Fasting blood was collected in early morning (07:30 to 09:00 hours) by the standard venipuncture procedure for biochemical measurements. Automated equipment was used to conduct the analyses.

Measurement of plasma glucose was performed by the enzymatic colorimetric glucose-oxidase/GOD-PAP method (Diasys, Holzheim, Germany), using the Vitalab Selectra 2 system (Elitech Group Solutions, Chicago, Illinois, USA). In this method, glucose is determined after enzymatic oxidation of glucose by glucose oxidase (Trinder reaction). Diabetes was categorized according to the American Diabetes Association (ADA, 2015) [12]. Fasting plasma-glucose levels ≥126 mg/dL or ≥100 and <126 mg/dL, respectively, are used to establish the diagnosis of diabetes and impaired fasting-plasma glucose (decreased glucose tolerance).

Insulin and cortisol levels were determined employing chemiluminescent microparticle immunoassay in the Architect-i1000SR Immunoassay Analyzer (Abbott, Illinois, USA). The cortisol assay has a sensitivity of ≤1 μg/dL and an assay precision of ≤ 10% total variation coefficient (VC) for serum samples. The reference values adopted for fasting cortisol in the mornings were 5.4–25.0 μg/dL or 149–690 nmol/L [12]. The sensitivity of the insulin test is ≤1.0 μU/mL and the precision is ≤ 7%. The reference values adopted for fasting insulin were 2.5–25.0 μU/mL or 17.9–179.3 pmol/L [12].

Insulin sensitivity was estimated from fasting-glucose and insulin data, using the homeostasis model assessment (HOMA) mathematical model: fasting insulin (μU/mL) + jejum glucose (nmol/L)/22.5 [13]. Insulin resistance was evaluated according to the body mass index (BMI) (1.2±0.65 for BMI <5, 1.8±0.98 for BMI 25–30, and 2.9±1.6 for BMI >30). The BMI was defined by dividing body weight by height squared and used for the classification of excess body weight. The cutoff level of 25 kg/m^2^ suggested by the World Health Organization (WHO) was employed.

Analyses of T cells (T helper/T cytotoxic lymphocyte ratio) were performed on a BD-FACS Calibur flow cytometer equipped with an argon laser and BD Multiset software for data analysis. The reagents employed were BD Multiset CD3/CD4/CD8/CD45 (Becton Dickinson TriTEST immunofluorescence, Becton Dickinson, San José, California, USA).

The overall antioxidant capacity was determined by Trolox equivalent antioxidant activity (TEAC), which was performed as described by Re et al. [14] to evaluate plasma non-enzymatic antioxidants. This method for screening antioxidant activity is a decolorization assay applicable to both lipophilic and hydrophilic antioxidants, including flavonoids, hydroxycinnamates, carotenoids, and plasma antioxidants. The final results are expressed in micromoles per liter (μM/L). Reference values established for TEAC, considering regional ethnic variations and laboratory conditions, were 1.83–2.07 μM/L.

Data of cortisol levels, overall antioxidant capacity and T Helper/T Cytotoxic lymphocytes are expressed as median [range] with 25th and 75th percentiles. Significant differences among median were analyzed using Prism 6.0 software (GraphPad, San Diego, CA, USA), and identified by the Kruskal-Wallis test with the Dunn’s post hoc test. The Chi-square (χ^2^) test using the STATA Data Analysis and Statistical Software version 9.1 (Texas, USA) was used to compare values of the control group with the positive-culture group and a crude odds ratio (OR) and 95% confidence interval (CI) were calculated. All variables were expressed as absolute and relative frequencies. *P* values < .05 were considered significant.

**Fig 1:**
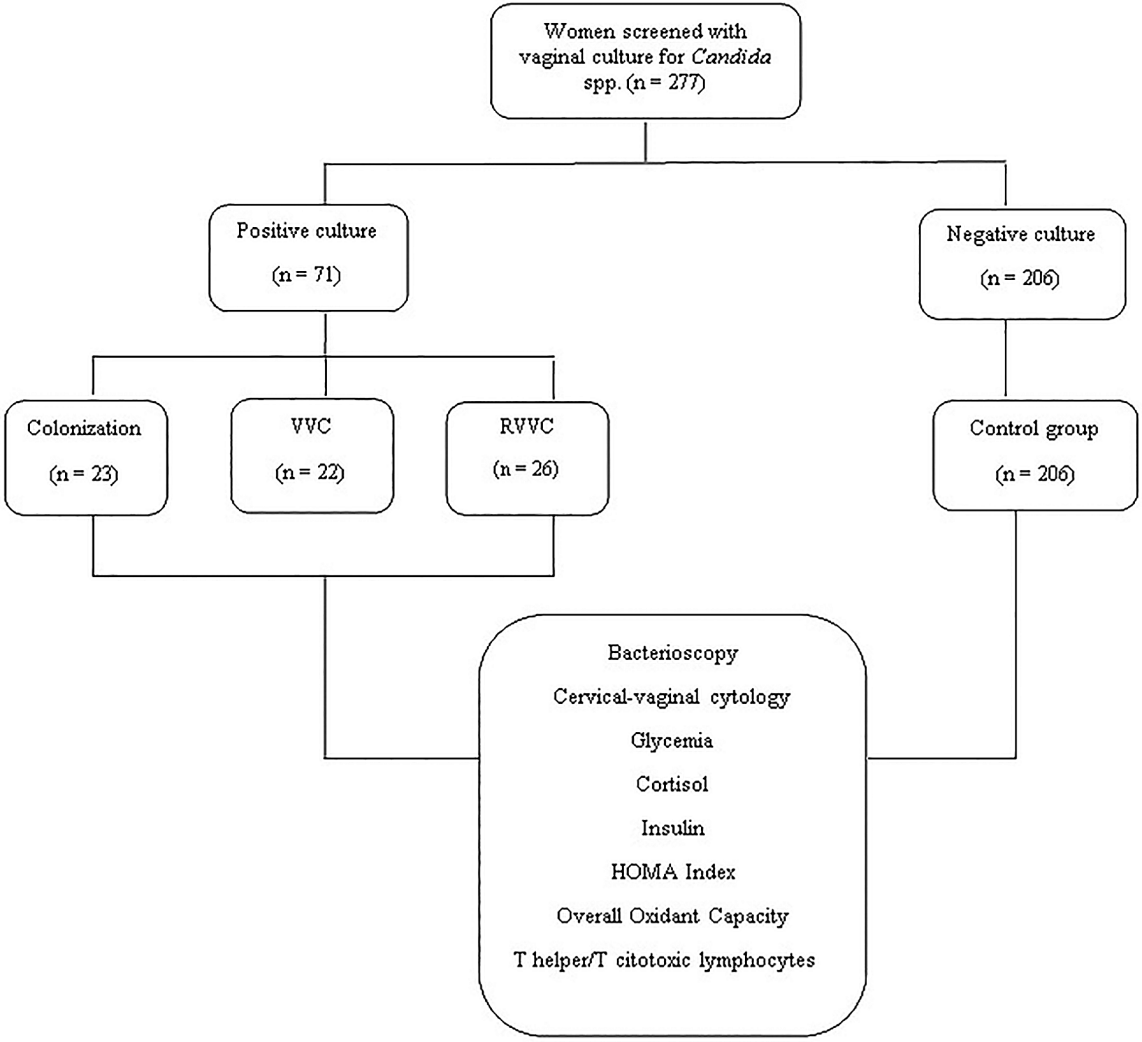
Flow chart of the study.

Fig 1 shows a flow chart of the study.

## Results

Table 1 shows the epidemiological characteristics of the 277 women (mean age 33.5±8.95 years) included in the study. Women who had two or fewer births and overweight women were significantly more associated with a positive culture for *Candida* spp. (OR 2.084; 95% CI 0.927–4.685; *p* = 0.049) and (OR 4.875; 95% CI 1.554–15.285; *p* = 0.002, respectively).

**Table 1:** Selected epidemiologic characteristics of 277 women who participated of study.

**Characteristics of women**	**Positive culture group**						**Control group**		**OR**	**[95% CI]**	*p value*
	**COL**		**VVC**		**RVVC**						
Age (years)	n	(%)	n	(%)	n	(%)	n	(%)			
14–30	12	52	6	27	9	35	81	39	0.702	0.374–1.318	0.269
31–40	8	35	10	46	11	42	59	29	0.510	0.249–1.046	0.061
>40	3	13	6	27	6	23	66	32	1.0		
Deliveries											
None	11	48	7	32	10	38	98	48	2.571	0.831–7.947	0.088
≤2	10	43	14	64	15	58	72	35	4.875	1.554–15.285	0.002*
>2	2	9	1	4	1	4	36	17	1.0		
Menarche (years)											
<12	5	22	8	36	11	42	47	23	1.0		
≥12	18	78	14	64	15	58	159	77	0.578	0.319–1.048	0.0679
Age first sexual intercourse											
Virgin	0	0	0	0	0	0	10	5	1.0		
≤16	10	43	6	27	7	27	57	58	4.035	0.471–34.516	0.167
>16	13	57	16	73	19	73	139	67	3.381	0.416–27.480	0.225
Hormonal Contraception											
No	13	57	13	59	14	54	116	56	1.001	0.580–1.726	0.996
Yes	10	43	9	41	12	46	90	44	1.0		
Number of sexual partners (last year)											
None	2	9	1	5	1	4	27	13	1.065	0.210–5.403	0.938
1	19	82	20	90	25	96	162	79	0.394	0.111–1.391	0.134
≥2	2	9	1	5	0	0	17	8	1.0		
BMI											
18–24	13	56	13	59	11	42	182	88	1.010	0.441–2.311	0.980
25–29	8	35	8	36	9	35	16	8	2.084	0.927–4.685	0.049*
≥30	2	9	1	5	6	23	8	4	1.0		

COL, vaginal colonization; VVC, vulvovaginal candidiasis; RVVC, recurrent vulvovaginal candidiasis; BMI, body mass index; OR, Odds Ratio; 95% CI, confidence interval.

*p<0 .05 was considered significant

Vaginal yeasts were isolated in 71 (25.6%) of the 277 women. Women with a negative culture composed the control group (n = 206; mean age 34±10.04 years). With respect to clinical profiles, 23 (32.4%) of the women with a positive culture were classified as COL (mean age 31±8.87 years), 22 (31.0%) as VVC (mean age 35±7.66 years), and 26 (36.6%) as RVVC (mean age 34±9.24 years). The mean ages were similar among women of all clinical profiles (*p* >0.05). Complete data on the isolation and identification of the yeasts were obtained from all 71 women with a positive culture, as follows: *C*. *albicans* (n = 66/71; 93%) and *Candida* non-*C*. *albicans* species (n = 5/71; 7%). *C*. *parapsilosis* was the most frequent *Candida* non-*C*. *albicans* species isolated (n = 3/71, 4.2%), followed by *C*. *tropicalis* and *C*. *glabrata* (n = 1, 1.4% each). *C*. *albicans* was the most frequently isolated yeast in all clinical profiles (*p* = 0.049; Table 2).

**Table 2:** Relative distribution of *Candida albicans* and *Candida* non-*C*. *albicans* species according to the clinical profile.

**Species**		Total (N = 71)		COL (N = 23)		VVC (N = 22)		RVVC (N = 26)		*p value*
		n	(%)	n	(%)	n	(%)	n	(%)	
*C*. *albicans*		66	93	19	82.7	21	95.5	26	100	0.049*
CNCA	*C*. *parapsilosis*	5 3	7 60	4 2	17.3 8.7	1 1	4.5 4.5	-	-	
	*C*. *glabrata*	1	20	1	4.3	-	-	-	-	
	*C*. *tropicalis*	1	20	1	4.3	-	-	-	-	
Total		71	100	23	100	22	100	26	100	

COL, vaginal colonization; VVC, vulvovaginal candidiasis; RVVC, recurrent vulvovaginal candidiasis. CNCA, *Candida* non-*C*. *albicans*.

*p<0.05 was considered significant

**Fig 2:**
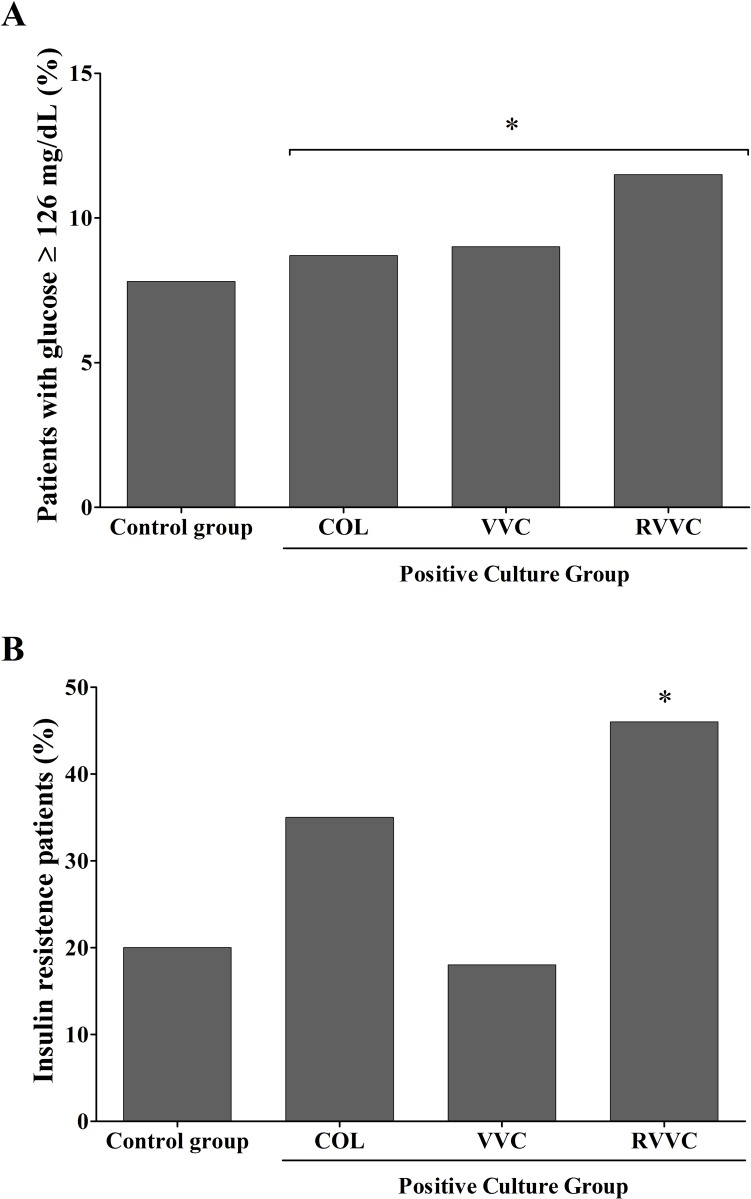
Abnormal glycemia and insulin resistance in 277 women studied. **A.** Percentage of women with abnormal glycemia (≥126 mg/dL) in control group compared to positive vaginal yeast culture group (colonized-COL, vulvovaginal candidiasis-VVC and recurrent VVC-RVVC). Positive culture group had more women with abnormal glycemia than control group (*p = 0.0002). **B.** Percentage of women with insulin resistance (HOMA Index ≥reference value according to body mass index-BMI) in positive vaginal culture group compared to control group. Positive culture group had more women with insulin resistance than control group (*p = 0.0002).

The mean values for glycemia were similar in all groups: control (83.40 ±12.47 mg/dL), COL (82±10.45 mg/dL), VVC (89.7±33.61 mg/dL), and RVVC (88.65± 15.9 mg/dL). Abnormal glycemia (≥100 mg/dL) was detected in 16 women (7.8%) of the control group, in 2 (8.7%) of the COL, in 2 (9%) of VVC, and in 3 (11.5%) of RVVC. The HOMA Index (HI) indicating insulin resistance was detected in 42 women (20%) of the control group, in 8 (34.8%) of the COL group, in 4 (18%) of VVC, and in 12 (46%) of RVVC. Compared to the control group, women with a positive culture (COL, VVC and RVVC combined) were associated with an abnormal glucose metabolism (OR 4.6; 95% CI 2.0–10.9; *p* = 0.0002; Fig 2A) and insulin resistance (OR 2.2; 95% CI 1.05–4.84; *p* = 0.00317; Fig 2B).

**Fig 3:**
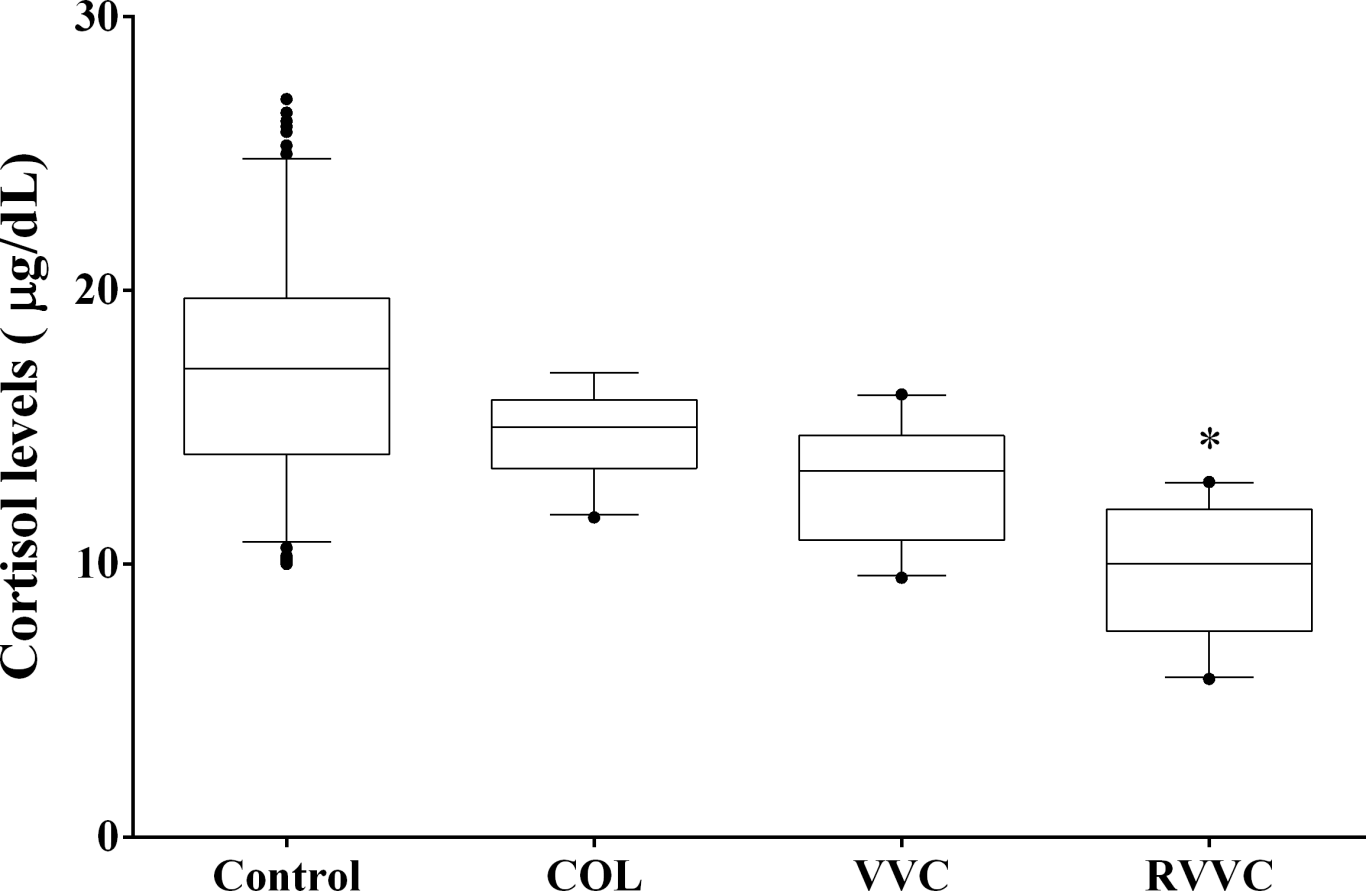
Morning cortisol levels according to the women clinical profiles. Median values of each group (Control, COL, VVC and RVVC) were calculated and compared using a Kruskal-Wallis and subsequently Dunn’s post-hoc test with all pairwise comparisons identified which groups differed. Box plots show the median, 25th and 75th percentiles of the cortisol levels. Whiskers extend to the 5th and 95th percentiles; filled circles represent outliers. Cortisol levels were determined in triplicate employing chemiluminescent microparticle immunoassay in the Architect-i1000SR Immunoassay Analyzer (Abbott, Illinois, USA). *p = 0.001, shows that women of the RVVC group has a lower mean cortisol level than the control and COL groups.

For early-morning cortisol levels, the median (25th-75th percentile) observed were: 17.15 μg/dL (14.00–19.73 μg/dL) in women of the control group, 15.00 μg/dL (13.50–16.00 μg/dL) of the COL group, 13.40 μg/dL (10.88–14.70 μg/dL) of the VVC group, and 10.00 μg/dL (7.55–12.00 μg/dL) of RVVC. Women of the RVVC group showed lower mean cortisol level than the control and COL groups (*p<* 0.001; Fig 3).

**Fig 4:**
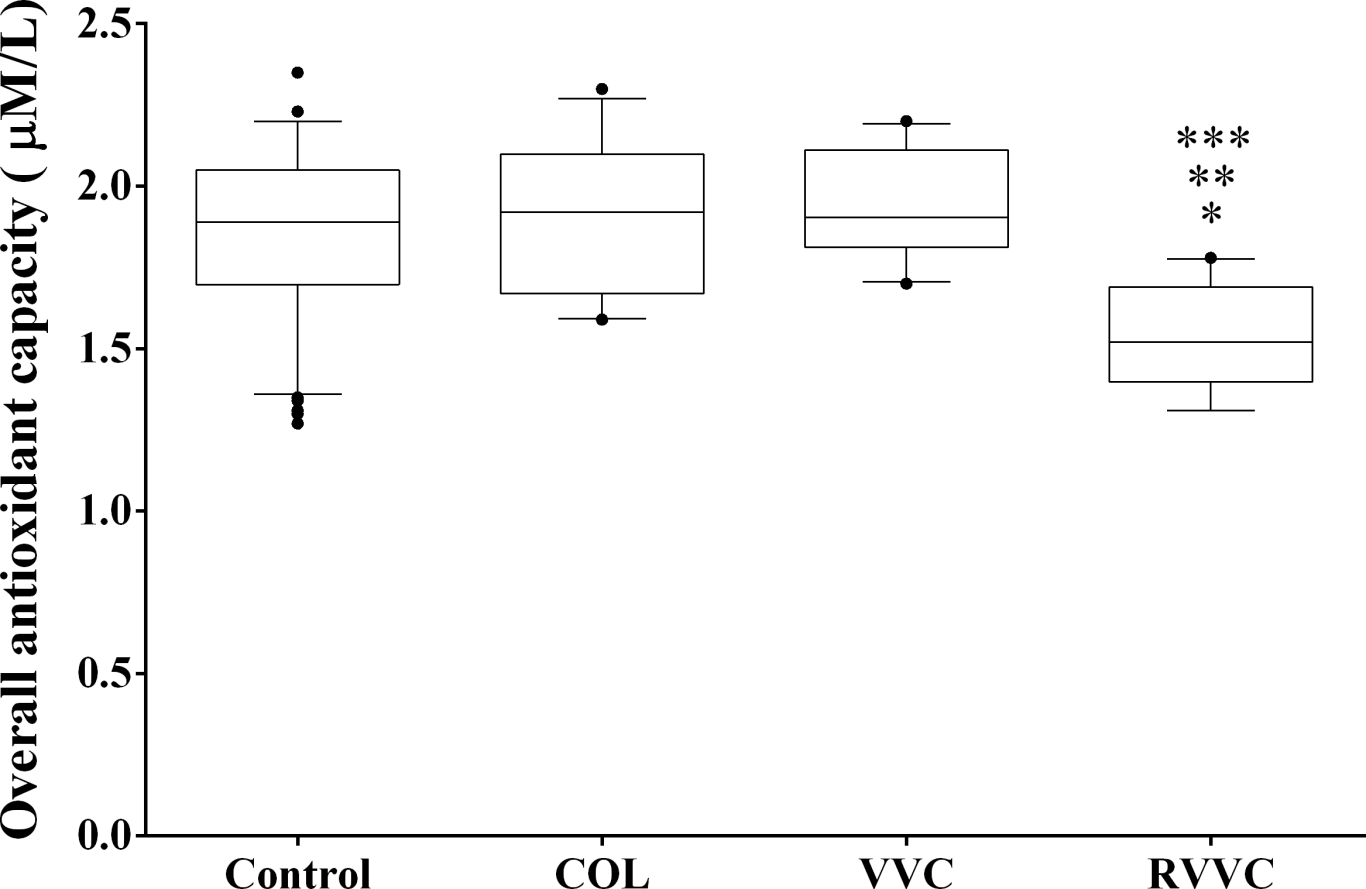
Overall antioxidant capacity according to the clinical profiles. Median values of each group (Control, COL, VVC and RVVC) were calculated and compared using a Kruskal-Wallis and subsequently Dunn’s post-hoc test with all pairwise comparisons identified which groups differed. Box plots show the median, 25th and 75th percentiles of the overall median level of antioxidant capacity. Whiskers extend to the 5th and 95th percentiles; filled circles represent outliers. The overall antioxidant capacity was determined in triplicate by Trolox equivalent antioxidant activity (TEAC), to evaluate plasma non-enzymatic antioxidants. Women with RVVC had significantly lower mean levels of antioxidant capacity than control (*p<0.0001), COL (**p<0.0001) and VVC (***p<0.0001) groups.

The overall median (25th-75th percentile) levels of antioxidant capacity were: 1.89 mM/L (1.69–2.05 mM/L) in the control group, 1.92 mM/L (1.67–2.10 mM/L) in the COL group, 1.90 mM/L (1.81–2.11 mM/L) in VVC, and 1.52 mM/L (1.39–1.69 mM/L) in RVVC. Women of the RVVC group had a significantly lower overall median level of antioxidant capacity than the control group (*p<*0.0001), the COL group (*p<*0.0001), and also VVC (*p<*0.0001; Fig 4).

**Fig 5:**
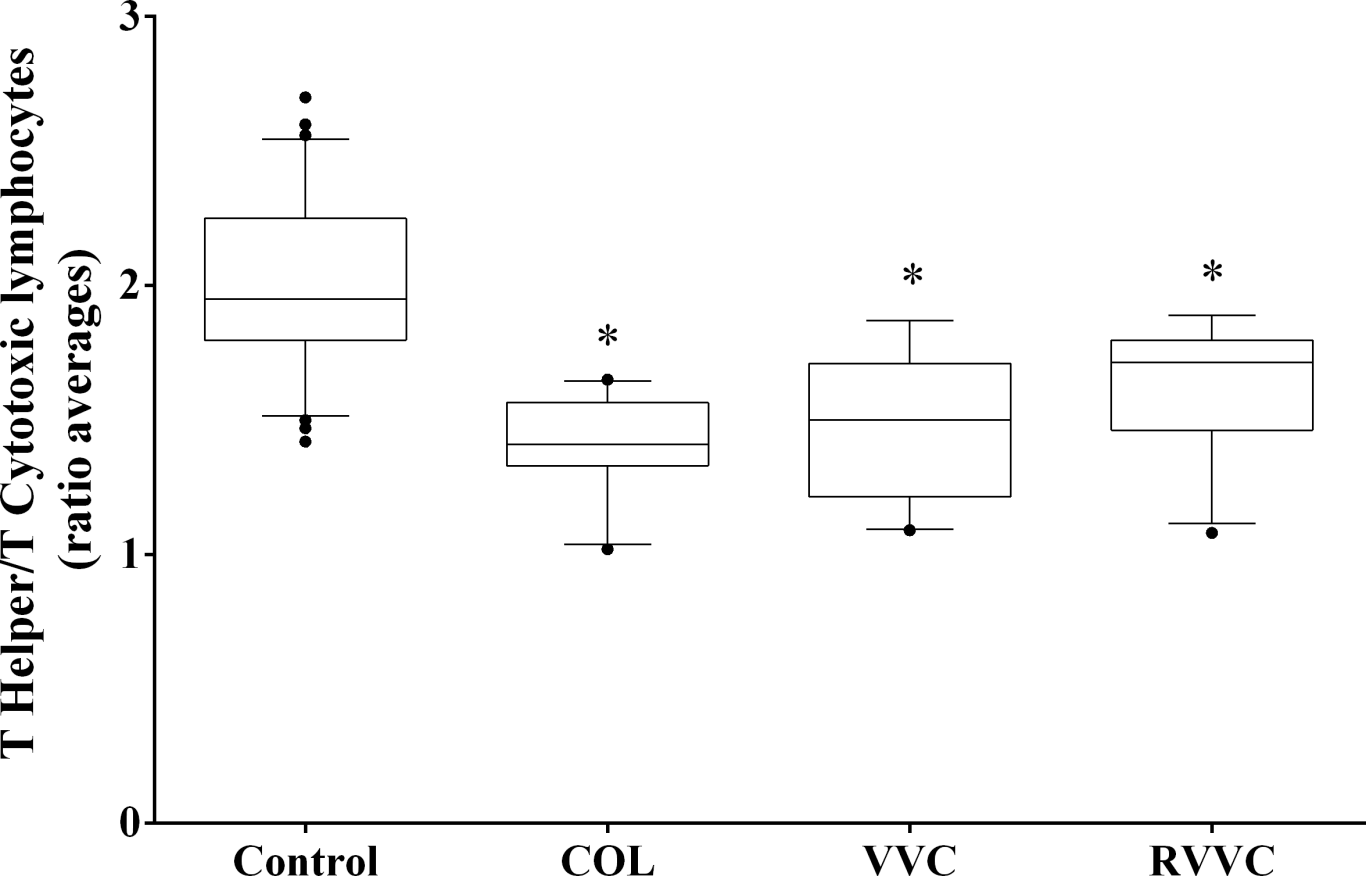
T helper/T cytotoxic lymphocytes ratio according to the clinical profiles. Median values of each group (Control, COL, VVC and RVVC) were calculated and compared using a Kruskal-Wallis and subsequently Dunn’s post-hoc test with all pairwise comparisons identified which groups differed. Box plots show the median, 25th and 75th percentiles of the overall median level of antioxidant capacity. Whiskers extend to the 5th and 95th percentiles; filled circles represent outliers. T helper/T cytotoxic lymphocytes ratio was determined in triplicate performed on a BD-FACS Calibur flow cytometer equipped with an argon laser and BD Multiset software for data analysis (Becton Dickinson TriTEST immunofluorescence, Becton Dickinson, San José, California, USA). *p<0.0001, the COL, VVC and RVVC group had lower T helper/T cytotoxic lymphocyte ratios than the control group.

The median (25th-75th percentile) T helper/T cytotoxic lymphocyte ratios were: 1.95 (1.79–2.25) in the control group, 1.41 (1.33–1.56) in COL, 1.50 (1.21–1.71) in VVC; and 1.71 (1.46–1.79) in RVVC. The COL, VVC and RVVC group had lower T helper/T cytotoxic lymphocyte ratios than the control group (*p*<0.0001 for all; Fig 5).

**Fig 6:**
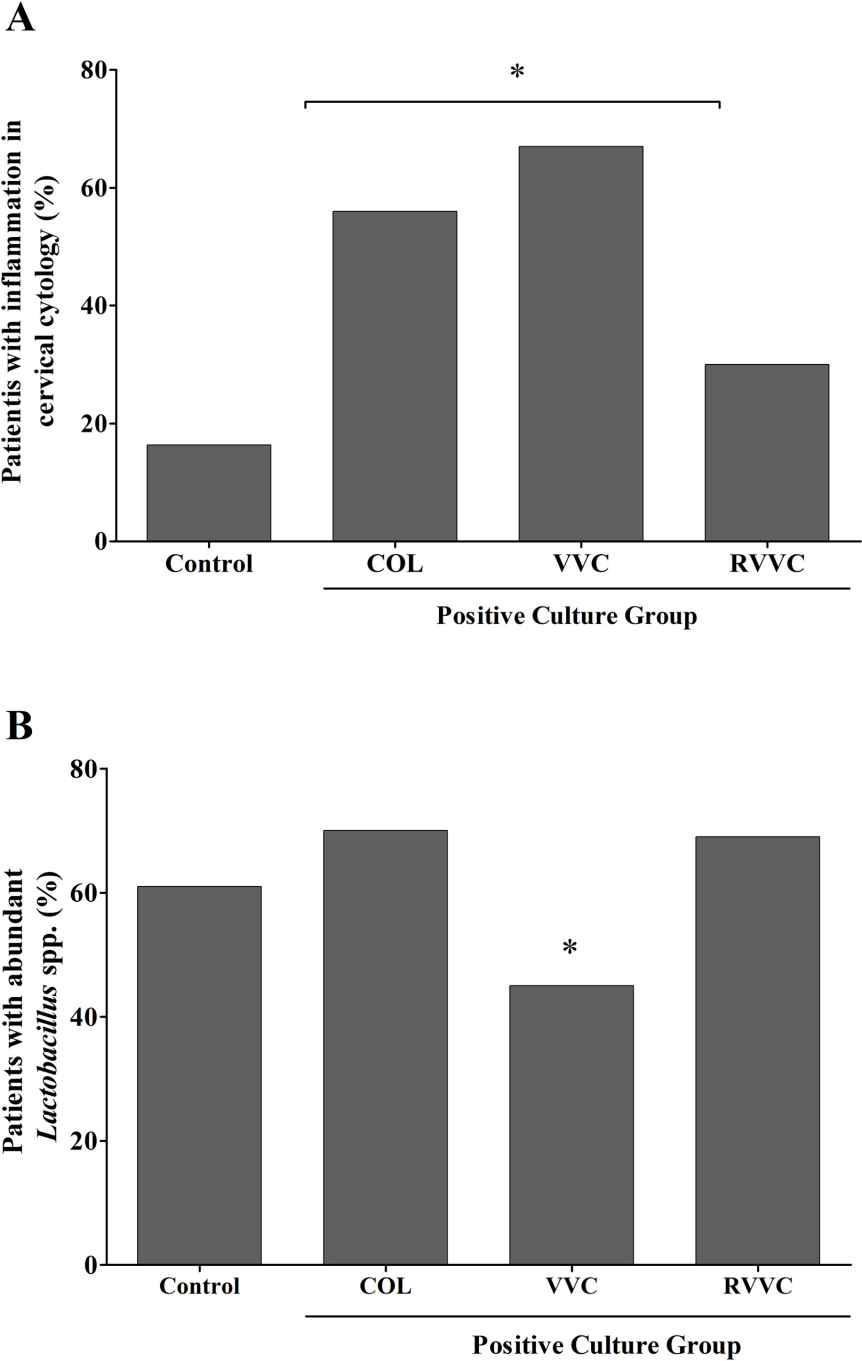
Cervical-vaginal cytology and bacterioscopy findings related to inflammation process and vaginal microbiota, respectively. A. Percentage of women with inflammatory processes demonstrated in microscopic analysis (400X) of vaginal cytology stained by Papanicolaou, according to clinical profiles. Samples were considered positive for inflammatory processes when smears showed leukocyte and cellular-inflammation characteristics of moderate and/or intense grade, in at least 20 different fields under optical microscopy at 400x magnification. Women with RVVC had a similar rates of vaginal inflammatory process in relation to women of control group (*p* = 0.188; OR = 1.96; 95% CI, 0.83–0.87%), while more women with VVC and COL had a vaginal inflammatory process than the control ones (*p = 0.0001; OR = 9.47; 95% CI, 4.03–16.44) and (*p = 0.0003; OR = 5.22; 95% CI, 1.97–8.69). B. Percentage of women with abundant vaginal *Lactobacillus* spp. by bacterioscopy, according to clinical profiles. Women with RVVC had no reduction in the quantities of *Lactobacillus* spp. compared to control group (*p* = 0.5223; OR = 1.429; 95% CI, 0.593–3.44).

Cytology of Pap showed a similar vaginal inflammatory process in women of the RVVC and control groups (OR 1.96; 95% CI 0.83–3.87; *p* = 0.188) while women of the VVC and COL groups showed a more-intense inflammatory process than the control group (OR 9.47; 95% CI 4.03–16.44; *p* = 0.0001) and (OR 5.22; 95% CI 1.97–8.69; *p* = 0.0003; Fig 6A).

Bacterioscopy showed that women in the COL and RVVC groups had similar normal *Lactobacillus* spp. vaginal microbiota to the control group (OR 1.429; 95% CI 0.593–3.44; *P* = .5223) and (OR 0.5896; 95% CI 0.2346–1.482; *p* = 0.2815, respectively). Women of the VVC group showed fewer vaginal lactobacilli compared to women of the COL and RVVC groups (OR 2.914; 95% IC 0.8634–9.836; *p* = 0.0404, and OR 2.712; 95% CI 0.8275–8.810; *p* = 0.0480, respectively; Fig 6B).

## Discussion

In this study, the early-morning cortisol level and overall antioxidant capacity were lower in women with RVVC than in women of the control and COL groups. This suggests that chronic stress and reduced antioxidant capacity may be specific predisposing factors related to the pathogenesis of RVVC. In addition, other factors such as diabetes mellitus and insulin resistance were related to positive vaginal yeast culture but not related to a specific clinical profile (COL, VVC and RVVC). These data support our hypothesis concerning the importance of chronic stress and reduced antioxidant capacity as specific predisposing factors for the development of RVVC.

Attenuated levels of morning cortisol and hyporesponsiveness of the hypothalamus-pituitary-adrenal axis have been shown to be indicative of chronic stress [13–15]. Also, stress may increase the risk for impaired function of the immune processes [16]. Clinical observations and experimental findings have further emphasized the role of chronic stress as an important triggering factor of many pathologies including atopic dermatitis, **s**ystemic lupus erythematosus, asthma, allergic rhinitis and *Candida* infections [13–17]. These studies are in agreement with our results, which indicated a relationship between chronic stress and vulvovaginal candidiasis (RVVC and VVC). Despite these results being in line with earlier findings that correlate lower levels of early-morning cortisol with RVVC [17, 18], it is possible that stress may be a predisposing factor for RVVC as well as a response to repeated infections.

A reduced overall antioxidant capacity has also been associated with immune deficiency, since its mechanism of action seems to be the modulation of signal transduction factors involving transcribing cells and immune-mediated cytokine production [19,20]. Additionally, it is important to consider that the elimination of *Candida* is a cell-mediated immune responses by the recruitment and activation of neutrophils, which respond to a proinflammatory cytokine [19–21]. The reduced overall antioxidant capacity, and the altered immune status in the RVVC group compared to all other groups, suggest that impaired immunity plays a role in the pathogenesis of RVVC.

Health benefits have also been attributed to antioxidants, including a reduced risk of coronary vascular diseases and some types of cancer [22]. In their etiology, these chronic diseases seem to result from cellular oxidative damage, caused by pro-oxidative agents such as free radicals, affecting lipids, proteins and DNA [7,8]. Dietary antioxidants may protect against these oxidative events in the body in cases where insufficient levels of endogenous antioxidants cannot counteract the reactive species [22]. It is possible that an increase in antioxidant defense provided by dietary vitamins and minerals may prevent RVVC.

In this study, we assessed the immune status by determining the lymphocyte T helper/T cytotoxic ratio in blood. Our results showed that women with RVVC had reduction in systemic cell-mediated immunity (CMI) responsiveness and the cytological findings indicated that women with RVVC had a lower local inflammatory response than the women in other groups with a positive culture (Fig 6). These data are in accordance with the findings of Fidel et al. [23] who showed that a local rather than a systemic CMI represents important host-defense mechanisms in the vaginal mucosa. Thereby, additional efforts and studies with the local T-cell count, besides other strategies such as interleukin genotyping, will be needed to assess the CMI responsiveness of patients with RVVC.

Diabetes mellitus has long been considered as one of the factors causing *Candida* vaginitis, mainly due to the changes induced by hyperglycemia, including decreased random motion of neutrophils, chemotaxis, phagocytosis, and microbial death [24–26]. High blood glucose levels promote yeast attachment and growth, and also interfere with immune responses in the host [27]. However, in many cases there is a lack of reliable and clinically relevant information proving diabetes mellitus as a risk factor to RVVC [28]. We recently showed that diabetes mellitus type 2 in Brazilian women was associated with vaginal yeast colonization, VVC and RVVC [29]. Donders et al [26] found impaired glucose tolerance in women with RVVC, compared to controls. However, we found no relationship between RVVC and diabetes mellitus. One hypothesis that partly explains this is that the increased glucose at the vaginal level could be more important than hyperglycemia in the pathogenesis of RVVC, because it would increase the adhesion and growth of yeasts as previously showed [26]. Still, Nyirjesy et al. [24] proposed that women with type 2 diabetes mellitus are at increased risk for vaginal *Candida* colonization because of glucosuria, which may also to justify our results. However, as we did not evaluate the vaginal and urinary glucose levels, additional studies are needed to examine such hypothesis.

The findings of studies on the impact of vaginal lactobacilar microbiota on the control of VVC and RVVC are contradictory [30,31]. Our results suggest that the vaginal communities of women with RVVC did not have reduced proportions of lactobacilli. These findings are consistent with others that also showed, using culture-dependent methods, that the development of RVVC was not correlated with an abnormal vaginal microbiota [31].

Finally, we are making a substantial assumption that both chronic stress (decreased early-morning cortisol levels) and decreased overall antioxidant capacity are present in women with RVVC. In addition, we have considered these data as host predisposing factors for developing recurrent status. In fact, our findings provided important information to better understand this pathology and lead to improved care for women with RVVC. However, this speculation was made based in a cross-sectional study, and this kind of design implies a limitation regarding to statements made. Despite these data alone are unable to reach results that would qualify as definitive, they supply a strong possibility. For a definitive conclusion, a longitudinal study, where women’s cortisol levels be tested during an infection process, following the treatment, and in periods of remission, must be carried on.

**Financial disclosure**: The authors did not report any potential conflict of interest.
